# Development of a Click Beetle Luciferase Reporter System for Enhanced Bioluminescence Imaging of *Listeria monocytogenes*: Analysis in Cell Culture and Murine Infection Models

**DOI:** 10.3389/fmicb.2017.01797

**Published:** 2017-09-26

**Authors:** Sadeeq Ur Rahman, Michael Stanton, Pat G. Casey, Angela Spagnuolo, Giuliano Bensi, Colin Hill, Kevin P. Francis, Mark Tangney, Cormac G. M. Gahan

**Affiliations:** ^1^APC Microbiome Institute, University College Cork, Cork, Ireland; ^2^School of Microbiology, University College Cork, Cork, Ireland; ^3^College of Veterinary Sciences and Animal Husbandry, Abdul Wali Khan University Mardan, Mardan, Pakistan; ^4^Cork Cancer Research Centre, University College Cork, Cork, Ireland; ^5^GSK Vaccines S.r.l., Siena, Italy; ^6^PerkinElmer, Alameda, CA, United States; ^7^SynBio Centre, University College Cork, Cork, Ireland; ^8^School of Pharmacy, University College Cork, Cork, Ireland

**Keywords:** *Listeria monocytogenes*, click beetle luciferase, bioluminescence, *in vivo* imaging, pathogen, virulence, clinical

## Abstract

*Listeria monocytogenes* is a Gram-positive facultative intracellular pathogen that is widely used as a model organism for the analysis of infection biology. In this context, there is a current need to develop improved reporters for enhanced bioluminescence imaging (BLI) of the pathogen in infection models. We have developed a click beetle red luciferase (CBR-*luc*) based vector (pPL2CBR^opt^) expressing codon optimized CBR-*luc* under the control of a highly expressed Listerial promoter (P_HELP_) for *L. monocytogenes* and have compared this to a *lux*-based system expressing bacterial luciferase for BLI of the pathogen using *in vitro* growth experiments and *in vivo* models. The CBR-*luc* plasmid stably integrates into the *L. monocytogenes* chromosome and can be used to label field isolates and laboratory strains of the pathogen. Growth experiments revealed that CBR-*luc* labeled *L. monocytogenes* emits a bright signal in exponential phase that is maintained during stationary phase. In contrast, *lux*-labeled bacteria produced a light signal that peaked during exponential phase and was significantly reduced during stationary phase. Light from CBR-*luc* labeled bacteria was more efficient than the signal from *lux*-labeled bacteria in penetrating an artificial tissue depth assay system. A cell invasion assay using C2Bbe1 cells and a systemic murine infection model revealed that CBR-*luc* is suited to BLI approaches and demonstrated enhanced sensitivity relative to *lux* in the context of *Listeria* infection models. Overall, we demonstrate that this novel CBR reporter system provides efficient, red-shifted light production relative to *lux* and may have significant applications in the analysis of *L. monocytogenes* pathogenesis.

## Introduction

*Listeria monocytogenes* is an opportunistic facultative intracellular pathogen which is capable of withstanding harsh environmental conditions, including stresses encountered during the processing, packaging and storage of foods (Gahan and Hill, 2005). Following consumption of contaminated foods *L. monocytogenes* survives the conditions encountered in the gastrointestinal tract and can progress to systemic infection (listeriosis) (Gahan and Hill, 2014). In humans, the clinical manifestations of listeriosis include meningitis, encephalitis, and septicemia predominately in the immune-compromised host, or late-term spontaneous abortion in pregnant individuals. The mortality rate can reach 30% during common-source outbreaks in susceptible hosts (Mead et al., 2000). *L. monocytogenes* infection in mice has been used as an important model for the analysis of intracellular parasitism and subsequent immunity and this research has benefited from the development of molecular tools to analyze bacterial and host responses *in vivo* (Cossart and Toledo-Arana, 2008; Lebreton et al., 2016).

Bioluminescence imaging (BLI) is a non-invasive technology that permits the molecular analysis of cells through the expression of proteins that emit visible light (Byrne et al., 2013). The development of sensitive light detection systems has permitted the use of BLI for the visualization of cell activity or localization in small animal models and other model systems (Contag and Bachmann, 2002). The technology is particularly useful for the *in vivo* tracking of bacterial infectious agents in real time in small animal models (Cronin et al., 2012a,b). Engineering of bacteria to produce light has typically involved cloning and expression of a gene system (the bacterial *lux* system) that is naturally found in species of *Vibrio* and *Photorhabdus* (Gahan, 2012). However, eukaryotic luciferases from natural sources (e.g., firefly, click beetle, sea pansy) can also be expressed in bacteria and may offer alternatives to the use of bacterial Lux for bacterial imaging studies (Gahan, 2012; Tangney and Francis, 2012; Chang et al., 2014; Karimi et al., 2016).

The bacterial *lux*-based bioluminescent reporter systems are based on the expression of the bacterial *lux* operon (*luxCDABE*) to produce light (Belkin et al., 1996). This system does not require the addition of substrate as the LuxCDE proteins generate endogenous substrate in the bacterial cytoplasm (Gahan, 2012). In contrast, the use of eukaryotic bioluminescence systems (Luc enzymes) requires the addition of exogenous luciferin as substrate (Gahan, 2012; Vorobyeva et al., 2015). The emission spectra of these systems also varies, with the insect luciferase enzymes emitting light in the green-red range of the visible light spectrum, compared with the bacterial luciferase system that emits blue-green light (Contag and Bachmann, 2002). This has implications for whole body imaging experiments, as blue-green light is of a relatively short wavelength (400–490 nm) and therefore has a reduced ability to penetrate host tissues compared with light emitted in the red region of the spectrum (Rice et al., 2001; Zhao et al., 2005).

To date, BLI vectors utilized in *L. monocytogenes* have successfully exploited the bacterial *lux* operon for *in vivo* localization studies (Hardy et al., 2004, 2009; Disson et al., 2008; van Pijkeren et al., 2010; Poulsen et al., 2011; Cronin et al., 2012a; Bergmann et al., 2013) and for *in situ* analysis of microbial gene expression (Riedel et al., 2009; Sleator et al., 2009; Joyce and Gahan, 2010; Quereda et al., 2016). In the current study, we created a bioluminescence reporter system based upon a red-optimized click beetle luciferase enzyme (CBR-*luc*) for use in *L. monocytogenes* and compare this with a previously described *lux* system (Riedel et al., 2007). The CBR-*luc* system utilizes a *Listeria*-specific chromosomal integration vector (pPL2) (Lauer et al., 2002) and constitutive expression of the click beetle luciferase was achieved using the highly expressed listerial synthetic promoter (P_HELP_) described previously (Riedel et al., 2007). We envisage that this vector will have significant applications in the study of *L. monocytogenes* in the context of both *in vivo* and *in vitro* experimental systems.

## Materials and Methods

### Strains, Media, and Bacterial Growth

The strains and plasmids used in this study are listed in **Table 1**. *E. coli* TOP10 (Invitrogen, Paisley, United Kingdom) were used as a cloning host for the creation of pPL2CBR^opt^. *E. coli* was grown aerobically at 37°C in Luria-Bertani broth (LB) medium. *L. monocytogenes* EGDe (Glaser et al., 2001) and murinized *L. monocytogenes* EGDe^m^ (Monk et al., 2010) were grown aerobically at 30°C in brain heart infusion (BHI) medium (Oxoid, Basingstoke, United Kingdom). Clinical strains of *L. monocytogenes* strains 130029, 140030, and 150008 were obtained from clinical cases of listeriosis in Ireland (manuscript in preparation). The pH of the LB and BHI medium was buffered with 100 mM 3-(*N*-morpholino)propanesulfonic acid (pH 7.4) where outlined in the text. When required, antibiotics were added to the media as follows: for *E. coli*; chloramphenicol (30 μg/ml), ampicillin (100 μg/ml) and for *L. monocytogenes*; chloramphenicol (7.5 μg/ml). For measurement of growth curves, *L. monocytogenes* strains were grown overnight and diluted in fresh medium until the OD_600_ reached 0.05. Subsequently strains were grown shaking at 30°C and readings were taken using a spectrophotometer. To measure bioluminescence during growth over time, overnight cultures were diluted in fresh medium to OD_600_ 0.05 and subsequently grown at 30°C. At selected time points, 50 μl of culture was mixed with 50 μl luciferin (PerkinElmer) with a final concentration of 150 μg/ml as described by the manufacturer.

**Table 1:** Strains and plasmids used in this study.

Strain/plasmid	Features	Source
*E. coli* TOP10	Cloning host	Invitrogen
*L. monocytogenes* strains		
EGDe	Wild type of serotype 1/2a with known genome sequence	Glaser et al., 2001
Murinized EGDe^m^	Mouse adapted EGDe strain of serotype 1/2a expressing modified internalin A (InlA) protein	Monk et al., 2010
EGDe::pPL2lux	A wild type EGDe strain integrated with pPL2lux vector at tRNA^Arg^ locus, which is a vector backbone and carrying Cm^r^ marker	Bron et al., 2006
EGDe::pPL2luxP_HELP_	A wild type EGDe strain with pPL2luxP_HELP_ integrated at tRNA^Arg^ expressing bacterial lux operon under the influence of a listerial highly expressed promoter (P_HELP_) and carrying Cm^r^ marker	Riedel et al., 2007
EGDe::pPL2CBR^opt^	A wild type EGDe strain with pPL2CBR^opt^ integrated at tRNA^Arg^ expressing click beetle red luciferase under the influence of a listerial highly expressed promoter (P_HELP_) and carrying Cm^r^ marker	This study
*L. monocytogenes*-4b-130029	A clinical strain of serotype 4b isolated from the CSF of a female patient with pPL2CBR^opt^ integrated at tRNA^Arg^ expressing click beetle red luciferase under the influence of a listerial highly expressed promoter (P_HELP_) and carrying Cm^r^ marker	This study
*L. monocytogenes*-4b-140030	A clinical strain of serotype 4b isolated from the CSF of a male patient with pPL2CBR^opt^ and integrated at tRNA^Arg^ expressing click beetle red luciferase under the influence of a listerial highly expressed promoter (P_HELP_) and carrying Cm^r^ marker	This study
*L. monocytogenes*-1/2a-150008	A clinical strain of serotype 1/2a isolated from the CSF of a male patient and integrated with pPL2CBR^opt^ integrated at tRNA^Arg^ expressing click beetle red luciferase under the influence of a listerial highly expressed promoter (P_HELP_) and carrying Cm^r^ marker	This study
Murinized EGDe::pPL2lux	A murinized wild type EGDe strain with pPL2lux vector integrated at tRNA^Arg^ locus, which is a vector backbone and carrying Cm^r^ marker	Bron et al., 2006
Murinized EGDe::pPL2lux P_HELP_	A murinized wild type EGDe strain with pPL2luxP_HELP_ integrated at tRNA^Arg^ expressing bacterial lux operon under the influence of a listerial highly expressed promoter (P_HELP_) and carrying Cm^r^ marker	Riedel et al., 2007
Murinized EGDe::pPL2CBR^opt^	A wild type EGDe strain with pPL2CBR^opt^ integrated at tRNA^Arg^ expressing click beetle red luciferase under the control of a listerial highly expressed promoter (P_HELP_) and carrying Cm^r^ marker	This study
PlasmidspPL2lux	A derivative of pPL2 listerial integrative vector backbone with PSA listeriophage integrase and attachment, Cm^r^ marker, bacterial *lux* operon without promoter.	Bron et al., 2006
pPL2lux P_HELP_	A derivative of pPL2 listerial integrative vector backbone with PSA listeriophage integrase and attachment site, bacterial lux operon with P_HELP_ promoter and Cm^r^ marker.	Riedel et al., 2007
pPL2CBR^opt^	A derivative of pPL2 listerial integrative vector backbone with PSA listeriophage integrase and attachment site and Cm^r^ marker.	This study

### Synthesis, Cloning, and Integration of CBR

**FIGURE 1:**
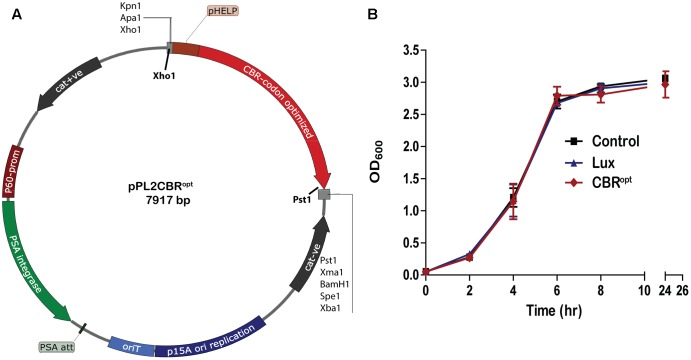
Cloning and expression of codon optimized click beetle luciferase. **(A)** Plasmid map of pPL2CBR^opt^ expressing codon-optimized click beetle luciferase (CBR^opt^) from the Listerial highly expressed promoter (P_HELP_) (Riedel et al., 2007) in a pPL2 backbone (Lauer et al., 2002). **(B)** Growth rate of *L. monocytogenes* EGDe tagged with a bacterial *lux* (Riedel et al., 2007), CBR^opt^ or an integrated vector control. Data and error bars represent mean and standard deviations, respectively, of triplicate samples. Each experiment was repeated at least three times in triplicate.

CBR was codon-optimized (CBR^opt^) for expression in *Listeria* and synthesized by GenScript (GenScript United States Inc., Piscataway, NJ, United States). The CBR^opt^ gene (Gene Accession No. KY628997) was synthesized with an intact upstream constitutive Listerial promoter, highly expressed Listerial promoter (P_HELP_) (Riedel et al., 2007) and the fragment was sub-cloned into the pPL2 backbone (Lauer et al., 2002) in *E. coli* using *Xho*1 and *Pst*1 restriction enzymes resulting in pPL2CBR^opt^ (see **Figure 1**). Plasmid DNA was isolated from *E. coli* using a QIAprep Spin Miniprep kit according to the manufacturer’s instructions (QIAGEN, Crawley, United Kingdom). The sequences were verified by sequencing through MWG Biotech AG (Ebersberg, Germany). Transformation of *L. monocytogenes* was performed as described (Park and Stewart, 1990). Successful integrants were screened for site-specific integration by PCR using template DNA of *L. monocytogenes* as described previously (Bron et al., 2006). Restriction endonucleases (Roche Diagnostic, Mannheim, Germany), T4 DNA ligase (Roche) and 2× PCR mixture (NEB, United Kingdom) were used as advised by the manufacturer. From each successful transformant one colony carrying the anticipated genotype was chosen for further bioluminescence analysis using an IVIS Lumina II system (PerkinElmer).

### *In Vitro* Tissue Depth Model

*Listeria monocytogenes* EGDe::pPL2CBR^opt^, *L. monocytogenes* EGDe::pPL2lux (silent vector) or *L. monocytogenes* EGDe::pPL2 luxP_HELP_ (expressing bacterial *lux* operon) (**Table 1**) were grown over night in BHI medium. Subsequently, cultures were grown in fresh BHI medium to a specified OD_600_. At specified OD_600_ (OD_600_ 1 or 1.5) cells were transferred to 96-well round bottom plates for imaging. Luciferin (PerkinElmer) was added to *L. monocytogenes* EGDe::pPL2CBR^opt^ (for *in vitro* 150 μg/ml, for *in vivo* 150 mg/kg body weight) according to the manufacturer’s instructions 15 min prior to imaging. Measurements of bioluminescence were repeated with intervening layers of uniformly cut meat slices (one to five layers, thickness varied from 2 to 10 mm depending on meat type) tested over the surface of the plate. Three different meat types (to reflect different physical and chemical parameters), chicken, serrano ham, and fresh sliced raw beef steak were tested. Images were acquired with up to 3 min exposure and processed by Living Image Software v4.2.

### Cell Invasion

All the cell culture procedures were performed as described previously (Bron et al., 2006). C2Bbe1 cells (CRC-2012; American Type Culture Collection), a clone of the Caco2 human adeno carcinoma cell line, were used for the cell invasion assay. Briefly, C2Bbe1 cells were maintained in Dulbecco’s modified Eagle’s medium (DMEM) with 4.5 g/l Glutamax (Gibco Laboratories, Grand Island, NY, United States), 10% fetal bovine serum (Gibco), 1% (vol/vol) non-essential amino acids (Gibco), 1% (vol/vol) penicillin–streptomycin (Gibco), and 0.01 mg/ml human transferrin (Calbiochem) at 37°C in a 5% CO_2_ environment. To perform the cell invasion assay, C2Bbe1 cells were trypsinized (Gibco), harvested by slow centrifugation at 500 × *g* for 5 min followed by resuspension in antibiotic free DMEM containing 10% fetal calf serum. Viable cells were counted using a standard trypan blue exclusion procedure. A known concentration of 1 × 10^5^ viable cells per well were seeded onto a 24- or 12-well flat bottom tissue culture plate (Sarstedt, Leicester, United Kingdom). Subsequently, the plates were incubated for 48–72 h at 37°C in a 5% CO_2_ atmosphere until confluent. Listeria strains were grown overnight in BHI medium, re-inoculated into BHI medium and grown until the OD600 reached 1.0 (1 × 10^9^ CFU/ml). Subsequently, bacterial cells were washed with antibiotic free DMEM medium twice and added to the C2Bbe1 cells at a multiplicity of infection of 100:1 followed by incubation at 37°C for 1 h in 5% CO_2_. The infection of C2Bbe1 cells was stopped after 1 h by adding gentamicin to a final concentration of 100 μg/ml, and cells were incubated further for 30 min to kill extracellular bacteria (Kim et al., 2004). Subsequently, the cells were washed three times with DMEM, and the levels of bioluminescence were measured using the IVIS Lumina II system (PerkinElmer). Immediately after imaging, the monolayers were lysed with 1 ml of ice-cold distilled water containing 0.01% triton (Sigma-Aldrich, St. Louis, MO, United States). The lysate was serially diluted in PBS and bacteria were plated onto BHI agar plates to determine the number of viable intracellular bacteria as CFU/ml.

### Animals, Infection, and Image Acquisition

For *in vivo* experiment, 8- to 10-week-old female BALB/c mice (Envigo, United Kingdom) weighing approximately 18–21 g were housed in pathogen free conditions at 22°C with a 12/12 light cycle. Animals were randomly assigned to groups (*n* = 4) and provided with mouse chow and water *ad libitum*. Before experiments, mice were afforded an adaptation period of at least 7 days. For bacteria preparation and infection, overnight cultures of *L. monocytogenes* were pelleted by centrifugation (7000 × *g* for 5 min), washed twice with PBS, and used to infect mice (*n* = 24) intraperitoneally at 2 × 10^5^ CFU in a total of 200 μl of PBS (Rea et al., 2004). The mice were followed for 72 h for imaging with 24 h intervals as described. After 24-, 48-, and 72-h post-infection, the infected mice were imaged following injection of luciferin (PerkinElmer, Inc., Boston, MA, United States) according to the manufacturer’s instructions at a dose of 150 mg/kg body weight administered via IP route as appropriate or imaged directly (mice infected with *L. monocytogenes* EGDe::pPL2luxP_HELP_). After whole body imaging, mice were sacrificed, and post-mortem resected organs were imaged using the IVIS system. Images were acquired with up to 5 min exposure and processed by Living Image Software v4.2 or v4.3.1.

### Ethics Statement

All animal procedures were performed in accordance with the national ethical guidelines prescribed by the Health Products Regulatory Authority (HPRA). Protocols and all the murine experiments were approved by the animal ethics committee of University College Cork (AERR #2010/003 and #2012/015).

### Statistical Analysis

**FIGURE 2:**
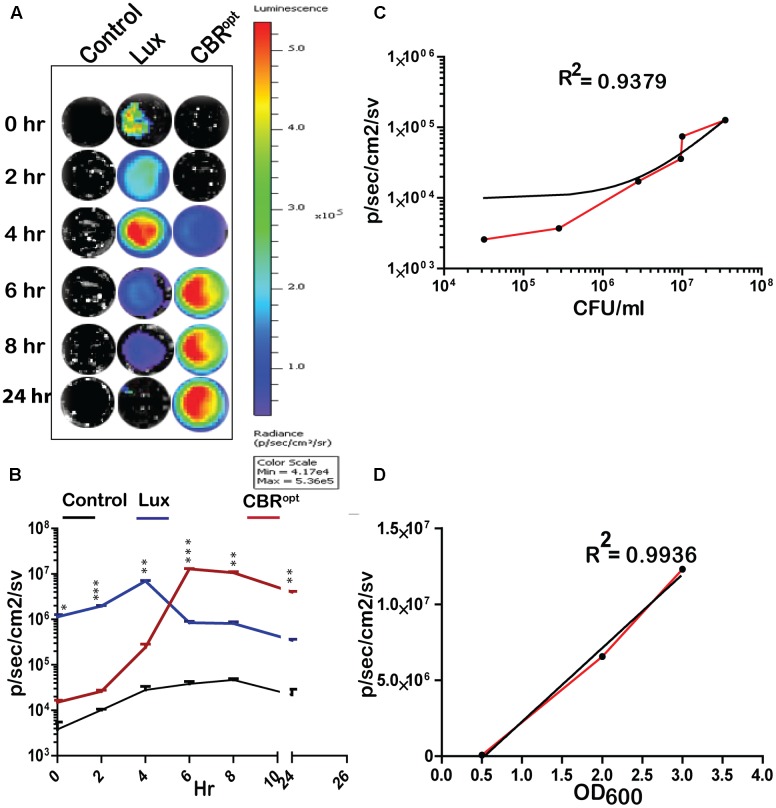
Luminescence of tagged *L. monocytogenes* during growth in media. **(A)** Luminescence imaging of luciferase-tagged *L. monocytogenes* strains. Luminescent images were taken using the PerkinElmer IVIS Lumina II system and processed using Living Image software as indicated in the text. The color bar represents bioluminescent signal intensity (in photons/s/cm^2^). **(B)** Luminescence (flux) of luciferase-tagged *L. monocytogenes* strains during *in vitro* growth in BHI broth. Data and error bars represent mean and standard deviation, respectively, of triplicate samples for each time point. A two-way ANOVA with Bonferroni post-test was used to compare groups. **(C)** Luciferase-tagged *L. monocytogenes* strains were grown to log phase and then serially diluted. Each dilution was imaged and also plated for CFU counts. The obtained data of total flux and CFU count was correlated. Statistical analysis showed a positive liner co-relation (with *r*^2^ = 0.94) between total flux emitted by CBR^opt^ and the luminescent bacterial. **(D)** *L. monocytogenes* EGDe::pPL2CBR^opt^ were grown in BHI medium and images were obtained by IVIS at given OD_600_ points. The data obtained was then analyzed that indicated a strong positive correlation between OD_600_ and total flux of the luminescent bacteria. Linear regression analysis was used to determine the relationship between flux and OD_600_ values in **(C,D)**.

All experiments were performed in triplicate at least two times. Data shown are for representative experiments. Statistical significance was based on either the Student’s *t*-test or ANOVA for comparison of groups (see **Figures 2B**, **6C**). GraphPad Prism 5 or IBM-SPSS v22 was used for graphs and statistical analysis. Linear regressions were carried out using GraphPad Prism 5. In all analysis, *p* < 0.05 was considered as significant.

## Results and Discussion

### Construction and Functional Analysis of a Click Beetle Red Luciferase Reporter System

The main purpose of the work reported herein was the construction and functional analysis of the pPL2CBR^opt^ bioluminescence vector (**Figure 1A**) using the backbone of the pPL2 integrating plasmid. pPL2 contains a PSA listeriophage integrase gene and attachment site that directs site-specific single copy integration into tRNA^Arg^ locus on the *L. monocytogenes* chromosome (Lauer et al., 2002). In our lab, we have previously successfully created pPL2-derivatives, pPL2lux and pPL2luxP_HELP_ that allow BLI through expression of the bacterial *lux* operon (Bron et al., 2006; Riedel et al., 2007). Here we used a similar approach to create a vector expressing a CBR-*luc* with potential applications in deep tissue whole body imaging.

For this purpose, a codon optimized CBR-*luc* (*CBR^opt^*) gene for optimal expression in *L. monocytogenes* was synthesized under the influence of a constitutive synthetic promoter, the highly expressed *Listeria* promoter P_HELP_ (Riedel et al., 2007). The synthesized construct was then sub-cloned into pPL2 using *Xho*1 and *Pst*1 restriction enzymes (**Figure 1A**). The sequence of the insert was verified and the novel vector was then transformed and integrated into the *L. monocytogenes* EGDe wild type strain and an engineered *L. monocytogenes* variant (EGDe^m^) expressing a murine-specific InlA (Monk et al., 2010). The pPL2 vector contains a PSA listeriophage integrase gene and attachment site that directs site-specific single copy integration into the tRNA^Arg^ locus on the *L. monocytogenes* chromosome (Lauer et al., 2002). The successful integrants were then examined for integration using PCR and positive clones were screened phenotypically for light emission. A total of 10 candidate integrants were picked and screened for integration with results indicating that 9 (90%) were positive integrants. In parallel, we used the pPL2luxP_HELP_ and pPL2lux (silent vector) (Riedel et al., 2007) as controls for integration and subsequent analysis of luminescence.

### CBR^opt^ Reporter Is Stably Integrated in *L. monocytogenes* and Does Not Impede Growth Rate

We tested the stability of the *L. monocytogenes* EGDe::pPL2 CBR^opt^ and EGDe^m^::pPL2CBR^opt^ strains in the absence of chloramphenicol. *L. monocytogenes* EGDe::pPL2CBR^opt^ and EGDe^m^::pPL2CBR^opt^ were sub-cultured for at least 50 generations in BHI medium without antibiotic selection. Dilutions of the last passage of the cultures were plated and the presence of the integrated plasmids in the resulting colonies was evaluated by scoring chloramphenicol resistant colonies. Our results indicated that all 50 colonies tested after 50 generations were found to retain chloramphenicol resistance indicating that the integrating plasmid was stably maintained without antibiotic selection pressure. Furthermore, at least 10 random chloramphenicol-resistant colonies were also tested phenotypically for light emission and the results indicated that all 10 tested colonies were bioluminescent.

Previous work has indicated that integration of *lux*-expressing pPL2 constructs does not influence bacterial growth rate relative to wild type bacteria (Riedel et al., 2007). In the current study we evaluated the growth behavior of *L. monocytogenes* EGDe::pPL2CBR^opt^ relative to EGDe::pPL2luxP_HELP_ (Riedel et al., 2007) and EGDe::pPL2 (Bron et al., 2006). Strains were grown in parallel under standard conditions (**Figure 1B**). Our data indicated that the growth of *L. monocytogenes* EGDe::pPL2CBR^opt^ was normal relative to the other strains tested (**Figure 1B**). Furthermore, the growth rates of *L. monocytogenes* EGDe::pPL2CBR^opt^ were also not affected in BHI (in which pH was adjusted to 7.4) or LB medium (data not shown).

### CBR^opt^ Can Be Used for Tagging Clinical *L. monocytogenes* Strains

*Listeria monocytogenes* EGDe is the most commonly used laboratory strain for the analysis of listerial pathogenesis (Glaser et al., 2001). However, there is increasing interest in the analysis of wild type clinical isolates in order to determine strain specific differences in pathogenesis (Maury et al., 2016). For this purpose, three clinical strains (**Table 1**), all originating from patients who suffered meningitis and isolated from cerebrospinal fluid (CSF) were transformed with pPL2CBR^opt^. Our results indicated that all three wild type clinical strains could be successfully tagged with our newly developed plasmid (**Supplementary Figure S1**). After 30 passages of these strains *in vitro* 50 colonies were tested and all retained chloramphenicol resistance indicating that the system is as stable as observed in the EGDe laboratory strain. We also determined that the *in vitro* growth rate of these wild type field isolates in BHI broth was not affected by the presence of the integrated plasmid (data not shown). We transformed the clinical isolates with pPL2luxP_HELP_ in order to provide a comparison with the CBR^opt^ system. Results indicated that bioluminescent profile followed the same light emission pattern as observed for the EGDe strain as described below (**Supplementary Figure S1**).

### CBR Signals Are Brighter and Produce Greater Flux Than *lux* During *In Vitro* Growth

We determined the bioluminescence profile of *L. monocytogenes* EGDe::pPL2CBR^opt^ over time. *L. monocytogenes* EGDe::pPL2 CBR^opt^ was grown in BHI broth for 24 h to monitor bioluminescence (**Figures 2A,B**). No bioluminescence was detected at the initial stage of growth. The bioluminescence profile increased as bacteria grew and bioluminescence reached a peak following 6 h of bacterial growth and remained relatively high during stationary phase. We directly compared the bioluminescence of *L. monocytogenes* EGDe::pPL2CBR^opt^ with the *L. monocytogenes* EGDe::pPL2luxP_HELP_ strain as both bioluminescent vectors are constructed using the pPL2 backbone with expression of luciferase under the same P_HELP_ promoter system (Riedel et al., 2007). Bioluminescence signals of bacterial luciferase (*L. monocytogenes* EGDe::pPL2luxP_HELP_) were higher during initial growth as compared to the isolate expressing CBR^opt^. However, the bioluminescence signal from *L. monocytogenes* EGDe::pPL2luxP_HELP_ diminished quickly as bacteria entered the stationary phase and the overall bioluminescence expression pattern for this strain was similar to our previously reported data (Riedel et al., 2007). A decrease in the bioluminescence signal during stationary phase may be a feature of light expression from the *luxABCDE* system as the phenomenon has also been described for other Gram-positive bacteria including *Streptococcus pneumonia* (Francis et al., 2001) and *Lactobacillus casei* (Oozeer et al., 2005). It is possible that the diminished Lux signal during stationary phase may be related to decreased metabolic recycling of FMN to FMNH_2_ (an essential component for bacterial Lux activity), and ATP production (Francis et al., 2001; Andreu et al., 2010) This hypothesis is partially supported by a previous study which demonstrated a 100-fold increase in signal after providing a gene (*frp*) encoding a NADPH-FMN oxidoreductase in-trans with *luxAB* enzymes in a yeast model system (Szittner et al., 2003). However, this limitation would not influence the activity of CBR, which in our system exhibits relatively robust activity in late exponential phase and stationary phase.

In order to measure light intensity per cell, bioluminescence signals were measured in parallel with determination of the total number of viable bacterial CFU. Our results indicated that CBR^opt^ signals were directly correlated with CFU (*r*^2^ = 0.9379; **Figure 2C**). A similar positive correlation was observed between the OD_600_ and total flux during growth in medium (**Figure 2D**). The strong positive correlation between CFU and total flux indicates that luminescence from CBR-*luc* tagged bacteria can successfully be used for accurate quantification of bacterial numbers during growth.

**FIGURE 3:**
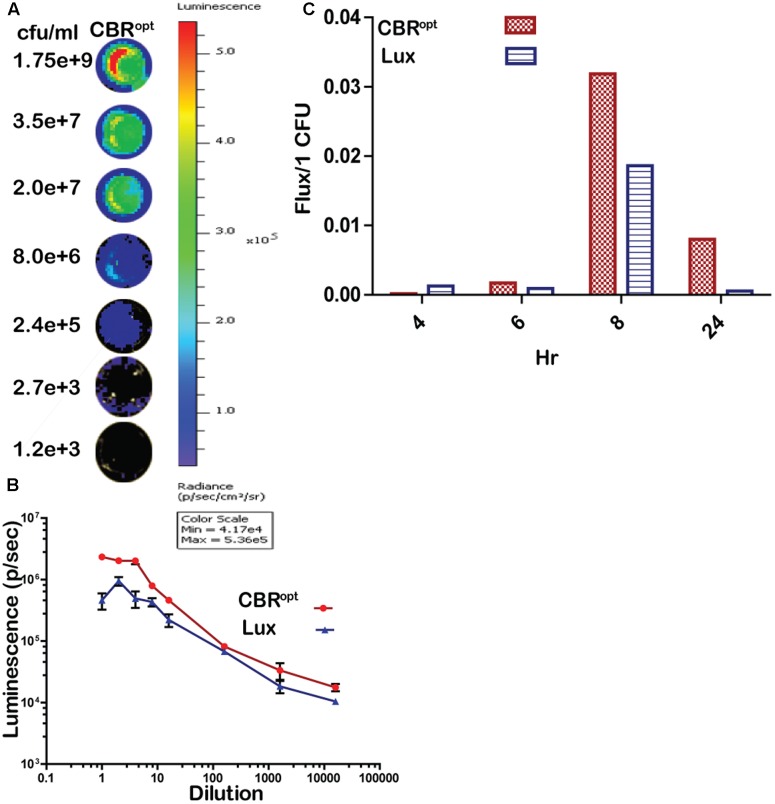
Luminescence per bacterium. **(A)** Luminescent images of CBR^opt^ tagged *L. monocytogenes* following imaging of specified numbers bacteria (indicated as CFU/ml). *L. monocytogenes* EGDe::pPL2CBR^opt^ were grown until mid-log phase. A 50-μl of culture in triplicate was then transferred to a 96-well plate and was serially diluted. Each dilution was imaged and also plated for CFU. A single representative well of the imaged bacterium is shown. **(B)** Correlation of the corresponding luminescence and CFU is shown. Data are expressed as total flux in photon/s. Data and error bars represent mean and standard deviation, respectively, of triplicate samples for each dilution. **(C)** CBR^opt^ or *lux*-tagged *L. monocytogenes* were grown in BHI medium. At given time points (4, 6, 8, and 24 h), 50 μl of culture in triplicate was then transferred to 96-well plate and was serially diluted. Each dilution was imaged and also plated for CFU counts to determine the total flux per CFU.

In order to directly compare signal intensity each luminescent strain was serially diluted until no luminescence signals were detectable (**Figures 3A,B**) and the CFU/ml of each representative dilution was calculated in order to determine relationship of total flux/CFU. The bioluminescence profile of both *lux* and CBR-*luc* luciferase reporters were indeed dependent on the number of the luminescent bacteria. Bioluminescence intensity was correlated with the number of bioluminescent bacteria both for EGDe::pPL2luxP_HELP_ and EGDe::pPL2CBR^opt^ during growth over time in BHI medium (**Figure 3B**) and the overall analysis indicated that CBR^opt^ produces greater flux/CFU when compared with bacterial luciferase during growth in medium (*p* > 0.05) (**Figure 3C**).

### Bioluminescence Profile of CBR^opt^ in an *In Vitro* Model of Varied Tissue Depth

**FIGURE 4:**
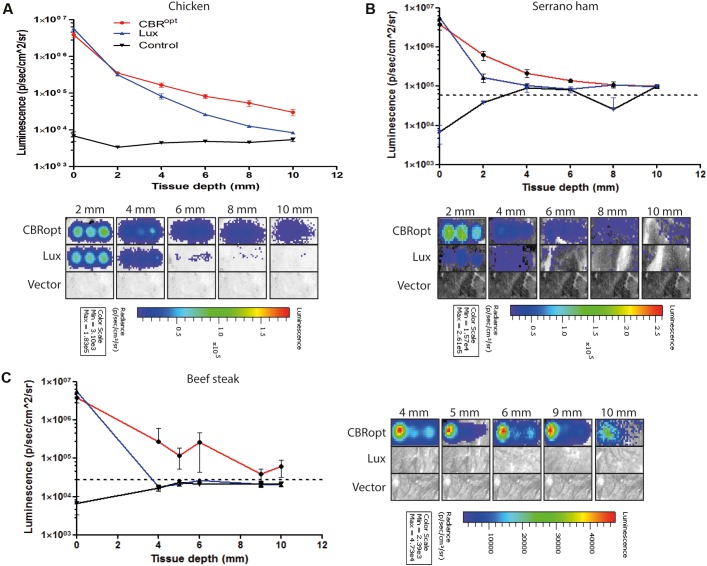
Ability of bioluminescence signals to penetrate an *in vitro* model tissue model. Luciferase-tagged and control (empty vector) *L. monocytogenes* strains were grown to log phase and were normalized to an identical OD_600_. A 50-μl of bacterial culture in triplicate was then transferred to a 96-well plate. Imaging was repeated with intervening layers of meat slices of various diameters. A representative experiment is shown. Attenuation of luminescence from bacterial luciferase is obvious in the red meat (beef steak) samples whereas CBR^opt^ labeled bacteria produced a signal that was clearly visible through layers of red meat as indicated. The Figure indicates the identical experiment carried out using slices of **(A)** chicken, **(B)** serrano ham, and **(C)** fresh sliced raw beef steak. In all cases, error bars represent mean ± standard deviation. The dotted line represents luminescence from meat placed over a well containing media alone.

CBR-*luc* produces light of a wavelength (∼600 nm) that is within the red region of the visible light spectrum. As red light has greater penetrating power in animal tissues, we predicted that light generated by CBR-*luc* would penetrate mammalian tissues more effectively than that of *lux* (in the blue spectrum at around 480 nm). We compared the ability of the CBR^opt^ and Lux luciferase signals to penetrate through varying depths of tissue using a previously described *in vitro* assay (Mason et al., 2016). Layers of cut meat slices were used to mimic varying levels of tissue depth. An aliquot of 50 μl of cells were deposited into a round bottom 96-well plate and light measurements were repeated following the addition of layers of meat slices to generate tissue depth as indicated in **Figure 4**. As expected a decrease in light detection was associated with an increase in tissue depth. Signal transmission and total flux of bacterial luciferase (*L. monocytogenes* EGDe::pPL2luxP_HELP_) quickly declined as the thickness of the tissue increased as compared to CBR^opt^ (*L. monocytogenes* EGDe::pPL2CBR^opt^). In particular *L. monocytogenes* EGDe::pPL2CBR^opt^ was clearly visible through multiple layers of raw beef steak whereas the penetrating power of light generated in *L. monocytogenes* EGDe::pPL2luxP_HELP_ was poor and below detectable limits (**Figures 4A,B**).

### BLI of CBR^opt^ during a Cell Invasion Assay

**FIGURE 5:**
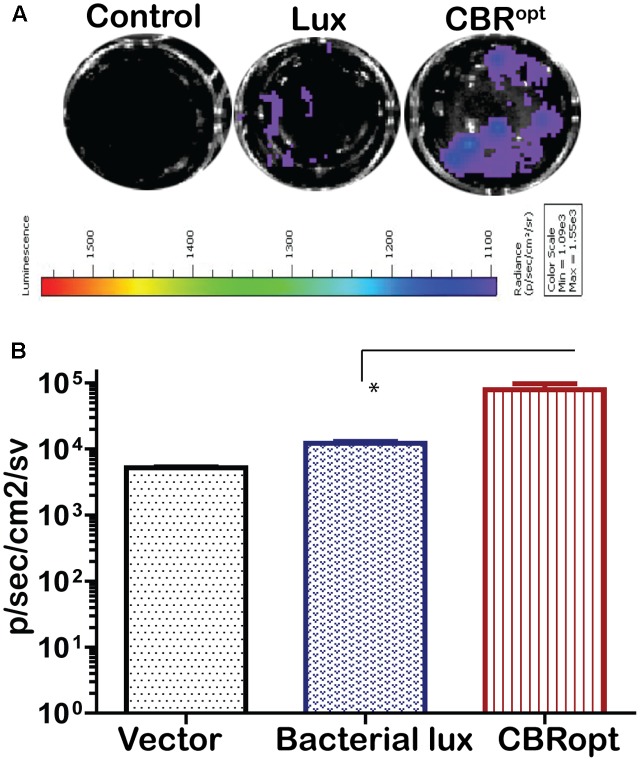
Comparison of luminescence during growth of *L. monocytogenes* in C2Bbe1 cell culture. **(A)** Luminescence images of luciferase-tagged *L. monocytogenes* or a control infecting C2Bbe1 cells after 1 h of infection. A 100 MOI infection rate was used. Cells were infected for about an hour, after that, extracellular bacteria were killed by gentamicin. The image was taken after washing superficial and non-invaded bacteria using. **(B)** Corresponding luminescence data of the luciferase-tagged *L. monocytogenes* or a control. CBR^opt^ clearly emit higher flux than the bacterial lux. Data and error bar represent mean and standard deviation, respectively, of the triplicate samples. ^∗^*p* < 0.05 based upon ANOVA analysis.

The pPL2luxP_HELP_ vector has previously proved effective for the imaging of *L. monocytogenes* infection in cultured mammalian cells (Riedel et al., 2007). In the current study, we investigated the bioluminescence profile of *L. monocytogenes* EGDe::pPL2CBR^opt^ during a cell invasion assay in order to determine the possibility of using this vector for monitoring and detection of EGDe in cell culture. For this purpose, *L. monocytogenes* EGDe::pPL2CBR^opt^, *L. monocytogenes* EGDe::pPL2luxP_HELP_ and *L. monocytogenes* EGDe::pPL2lux were used to infect C2Bbe1 cells for 1 h and the levels of bioluminescence and number of the intracellular bacteria were determined (**Figures 5A,B**). The efficacy of invasion did not differ between the strains and bacteria were isolated at similar levels from each well. However, the bioluminescence intensity indicated that *L. monocytogenes* EGDe::pPL2CBR^opt^ produced a consistently brighter signal and significantly higher flux than *L. monocytogenes* EGDe::pPL2luxP_HELP_ in the context of *in vitro* infection (**Figures 5A,B**). This suggests that the pPL2CBR^opt^ vector may have applications for BLI of *Listeria* during infection assays, perhaps with the potential for use in high throughput assay systems.

### CBR^opt^ Facilitates Robust *In Vivo* Whole Body Imaging of Murine Infection

Murine infection is widely used for the analysis of factors contributing to the pathogenesis of *L. monocytogenes* (Lecuit, 2007; Cossart and Toledo-Arana, 2008). A number of pathogens such as *Trypanosoma brucei* (Van Reet et al., 2014), *Mycobacterium tuberculosis* (Chang et al., 2014), and *Lactobacillus* species (Daniel et al., 2015) have been previously tagged with CBR reporters that provided enhanced whole body images in murine infection model. Given the improved signal intensity mediated by CBR during *in vitro* growth, in particular during late exponential and stationary-phase, we examined the signal intensity from *L. monocytogenes* EGDe::pPL2CBR^opt^ during murine infection. For this purpose, three groups of 12 mice were infected with murinized *L. monocytogenes* EGDe^m^::pPL2CBR^opt^, murinized *L. monocytogenes* EGDe^m^::pPL2luxP_HELP_, and murinized EGDe^m^::pPL2lux with a standard inoculum (2 × 10^6^ CFU/ml) via the intraperitoneal route. Four mice were left uninfected to serve as the control group. Four mice from each group were than imaged at 24 h intervals post-infection until 72 h. For the mice infected with *L. monocytogenes* EGDe^m^::pPL2CBR^opt^ luciferin was injected intraperitoneally 10 min prior to imaging. After imaging, mice were sacrificed and the resected organs (liver, spleen, and kidneys) were imaged for bioluminescence and further processed for counting the number of viable bacteria. Results of the whole body imaging of mice indicated that no bioluminescence was observed 24 h post-infection in any of the strains. No bioluminescence signals were detectable after dissecting the mice and imaging individual organs at this time point. This lack of bioluminescence correlated well with the lower number of viable bacteria recovered from the resected organs of the infected mice. Of these, the highest number of CFU was recovered from the spleen where CFU/ml were in the range of 1 × 10^3^.

**FIGURE 6:**
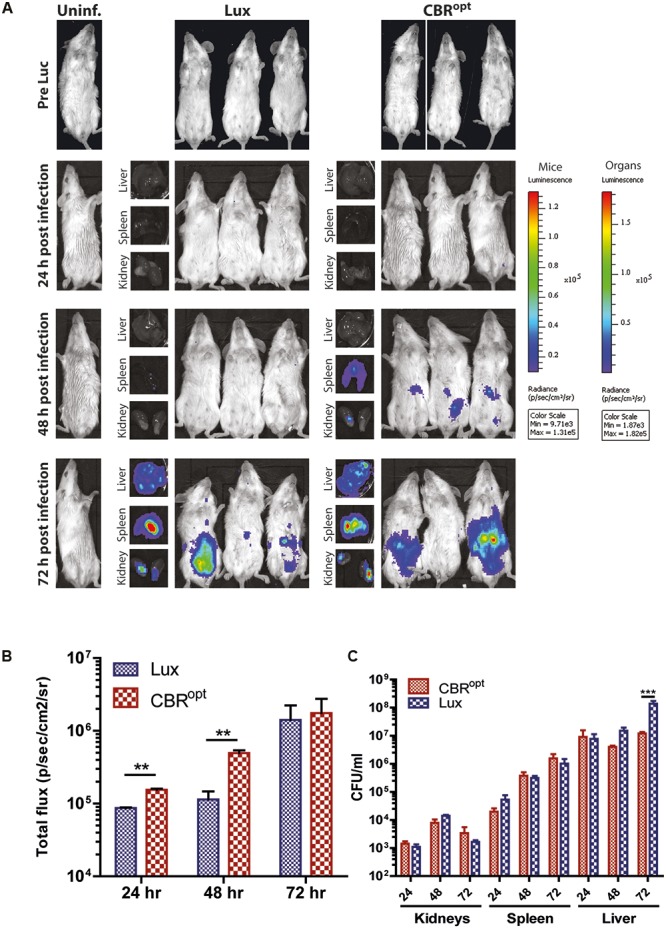
*In vivo* BLI. **(A)** *In vivo* BLI of sample mice infected with murinized *L. monocytogenes* EGDe tagged with either bacterial Lux or CBR^opt^. Mice (*n* = 4) were infected ip with *L. monocytogenes* at a dose of 2 × 10^6^ and the infection was followed over time: 24, 48, and 72 h. Post-mortem-resected kidneys, spleen, and liver are shown along with the corresponding mouse. Light emission from CBR^opt^ infected mice is clearly detectable at 48 h post-infection, unlike that of animal infected with the Lux-tagged strain. **(B)** Quantitative BLI readout from murine whole body imaging at given time points. Luminescence is shown as total flux (p/s/cm^2^/sv). **(C)** Bacterial count of post-mortem resected organs determined after plating homogenate. Data and error bars represent mean and standard deviation, respectively, of quadruplicate samples. Asterisks “^∗∗^” indicates *p* < 0.05 based upon Student’s *t*-test. ^∗∗∗^*p* < 0.01.

Notably, mice infected with *L. monocytogenes* EGDe^m^::pPL2CBR^opt^ produced detectable bioluminescence signals at 48 h post-infection, while no signals were detected from mice infected with *L. monocytogenes* EGDe^m^::pPL2luxP_HELP_ or the *L. monocytogenes* EGDe^m^::pPL2lux control. We acknowledge that there was some variability in observable signals between individual mice (when imaged at the specified sensitivity) as noted in previous studies by other groups (Poulsen et al., 2011). The number of CFU in these animals reached approximately 1 × 10^6^ CFU/ml in the spleen and liver. Importantly, there was no significant difference in CFU/ml among the infected strains recovered from the infected mice post-mortem in resected organs (results not shown). Interestingly, at 48 h infection bioluminescent signals were detected in the resected liver and spleen of mice infected with *L. monocytogenes* EGDe^m^::pPL2luxP_HELP_, but no signals were detectable during whole body imaging, indicating that these signals were attenuated during whole body imaging, potentially as a consequence of the low penetrating power of the shorter wavelength light emitted by Lux. In contrast, bioluminescence signals were observed during whole body imaging of mice infected with *L. monocytogenes* EGDe^m^::pPL2CBR^opt^ at 48 h post-infection in addition to a clear signal from post-mortem resected organs (**Figure 6**).

In a tandem study using infection with wild type (non-murinized) EGDe we also noted a clearly enhanced signal from mice infected with CBR^opt^ labeled bacteria relative to mice infected with Lux-tagged EGDe (**Supplementary Figure S2**). In this experiment, bacterial burdens in the internal organs were comparable between the groups though the infectious load in the organs was noticeably lower than mice infected with EGDe^m^ (not shown). Again this suggests that the signal from CBR^opt^ may provide greater sensitivity at low bacterial loads.

Overall, the ability to detect luminescence signals in mice infected with *L. monocytogenes* EGDe^m^::pPL2CBR^opt^ at 48 h suggested that pPL2CBR^opt^ provides for increased sensitivity in the context of whole body imaging. At higher bacterial numbers on day 3 of infection the difference in signal intensity in whole body imaging between the Lux and CBR-labeled bacteria was less evident (**Figure 6**).

## Conclusion

In conclusion, we have constructed a system for the stable chromosomal integration of constitutively expressed CBR luciferase in *L. monocytogenes*. In comparison to a *L. monocytogenes* strain expressing bacterial luciferase (LuxAB), CBR provides a greater luminescence signal during the stationary phase of growth and better penetrating power in an *in vitro* tissue depth model. We also noted enhanced sensitivity during murine infection and in an *in vitro* cell infection assay. A limitation of using CBR is the necessity to add luciferin substrate to the system. This adds an extra variable, as the availability of substrate in complex systems would certainly affect signal intensity. In contrast bacterial *lux* systems encode the capacity to generate endogenous substrate thereby potentially eliminating this extra variable. Given the different emission spectra of each system there is significant potential to utilize both in combination in order to measure dual bacterial populations in complex systems as has been described recently (Daniel et al., 2015). Overall, we suggest that the pPL2CBR^opt^ vector may have significant applications for the study of *L. monocytogenes* in various model systems.

## Author Contributions

SUR and CG wrote the paper. SUR, MS, PC, AS, GB, CH, KF, MT, and CG proofread the paper. SUR, MS, PC, AS, GB, CH, KF, MT, and CG designed experiments. SUR, MS, PC, and AS carried out experiments. SUR, MS, PC, AS, GB, CH, KF, MT, and CG analyzed experiments.

## Conflict of Interest Statement

AS and GB are permanent employees of the GSK group of companies. GB reports ownership of GSK restricted GSK shares. The other authors declare that the research was conducted in the absence of any commercial or financial relationships that could be construed as a potential conflict of interest.

## Supplementary Material

FIGURE S1 Bioluminescence profile of clinical listerial strains labeled with CBR^opt^ reporter. EGDe-4b-130029:pPL2CBR^opt^, EGDe-4b-140030::pPL2CBR^opt^, and EGDe-1/2a-150008::pPL2CBR^opt^ (see **Table 1**) were integrated with the newly developed codon optimized (CBR^opt^) reporter. The tagged strains were then grown in BHI medium and images were recorded at different time intervals. The overall bioluminescence profile is shown. Data and error bars represent mean and standard deviation, respectively, of triplicate samples for each time point. Asterisks “^∗∗^” indicates *p* < 0.05 based upon Student’s *t*-test.

FIGURE S2 *In vivo* BLI of mice infected with wild type *L. monocytogenes* EGDe. **(A)** Whole body luminescence image of BALB/c mice infected with wild type *L. monocytogenes* EGDe tagged with either bacterial Lux or CBR^opt^. Mice were infected ip with a dose of 2 × 10^5^ bacteria and were imaged for luminescence at 72 h post-infection. A clear and robust signal is detectable from mice infected with CBR^opt^ tagged bacteria at this timepoint. A luminescent signal that exceeded background (uninfected animals) was detected in every *L. monocytogenes* EGDe CBR^opt^ infected mouse. The number of CFU/ml recovered from infected mice were not significantly different between both groups (not shown). **(B)** Graph showing the signal intensity from the whole body images obtained at 72 h from infected mice. Luminescence is shown as total flux (p/s/cm^2^/sr). Data and error bars represent mean and standard error.

## References

[B1] Andreu N. Zelmer A. Fletcher T. Elkington P. T. Ward T. H. Ripoll J. (2010). Optimisation of bioluminescent reporters for use with mycobacteria. *PLOS ONE* 5:e10777 10.1371/journal.pone.0010777 PMC2875389 20520722

[B2] Belkin S. Smulski D. R. Vollmer A. C. Van Dyk T. K. LaRossa R. A. (1996). Oxidative stress detection with *Escherichia coli* harboring a katG’::lux fusion. *Appl. Environ. Microbiol.* 62 2252–2256. 8779563 10.1128/aem.62.7.2252-2256.1996 PMC168006

[B3] Bergmann S. Rohde M. Schughart K. Lengeling A. (2013). The bioluminescent *Listeria monocytogenes* strain Xen32 is defective in flagella expression and highly attenuated in orally infected BALB/cJ mice. *Gut Pathog.* 5:19 10.1186/1757-4749-5-19 PMC3720536 23856386

[B4] Bron P. A. Monk I. R. Corr S. C. Hill C. Gahan C. G. (2006). Novel luciferase reporter system for in vitro and organ-specific monitoring of differential gene expression in *Listeria monocytogenes*. *Appl. Environ. Microbiol.* 72 2876–2884. 10.1128/AEM.72.4.2876-2884.2006 16597994 PMC1449049

[B5] Byrne W. L. DeLille A. Kuo C. de Jong J. S. van Dam G. M. Francis K. P. (2013). Use of optical imaging to progress novel therapeutics to the clinic. *J. Control. Release* 172 523–534. 10.1016/j.jconrel.2013.05.004 23680286

[B6] Chang M. Anttonen K. P. Cirillo S. L. Francis K. P. Cirillo J. D. (2014). Real-time bioluminescence imaging of mixed mycobacterial infections. *PLOS ONE* 9:e108341 10.1371/journal.pone.0108341 PMC4180448 25265287

[B7] Contag C. H. Bachmann M. H. (2002). Advances in in vivo bioluminescence imaging of gene expression. *Annu. Rev. Biomed. Eng.* 4 235–260. 10.1146/annurev.bioeng.4.111901.093336 12117758

[B8] Cossart P. Toledo-Arana A. (2008). *Listeria monocytogenes*, a unique model in infection biology: an overview. *Microbes Infect.* 10 1041–1050. 10.1016/j.micinf.2008.07.043 18775788

[B9] Cronin M. Akin A. R. Collins S. A. Meganck J. Kim J. B. Baban C. K. (2012a). High resolution in vivo bioluminescent imaging for the study of bacterial tumour targeting. *PLOS ONE* 7:e30940 10.1371/journal.pone.0030940 PMC3266281 22295120

[B10] Cronin M. Stanton R. M. Francis K. P. Tangney M. (2012b). Bacterial vectors for imaging and cancer gene therapy: a review. *Cancer Gene Ther.* 19 731–740. 10.1038/cgt.2012.59 22996740

[B11] Daniel C. Poiret S. Dennin V. Boutillier D. Lacorre D. A. Foligne B. (2015). Dual-color bioluminescence imaging for simultaneous monitoring of the intestinal persistence of *Lactobacillus plantarum* and *Lactococcus lactis* in living mice. *Appl. Environ. Microbiol.* 81 5344–5349. 10.1128/AEM.01042-15 26025906 PMC4510179

[B12] Disson O. Grayo S. Huillet E. Nikitas G. Langa-Vives F. Dussurget O. (2008). Conjugated action of two species-specific invasion proteins for fetoplacental listeriosis. *Nature* 455 1114–1118. 10.1038/nature07303 18806773

[B13] Francis K. P. Yu J. Bellinger-Kawahara C. Joh D. Hawkinson M. J. Xiao G. (2001). Visualizing pneumococcal infections in the lungs of live mice using bioluminescent *Streptococcus pneumoniae* transformed with a novel gram-positive lux transposon. *Infect. Immun.* 69 3350–3358. 10.1128/iai.69.5.3350-3358.2001 11292758 PMC98294

[B14] Gahan C. Hill C. (2005). Gastrointestinal phase of *Listeria monocytogenes* infection. *J. Appl. Microbiol.* 98 1345–1353. 10.1111/j.1365-2672.2005.02559.x 15916648

[B15] Gahan C. G. (2012). The bacterial lux reporter system: applications in bacterial localisation studies. *Curr. Gene Ther.* 12 12–19. 10.2174/156652312799789244 22263920

[B16] Gahan C. G. Hill C. (2014). *Listeria monocytogenes*: survival and adaptation in the gastrointestinal tract. *Front. Cell. Infect. Microbiol.* 4:9 10.3389/fcimb.2014.00009 PMC3913888 24551601

[B17] Glaser P. Frangeul L. Buchrieser C. Rusniok C. Amend A. Baquero F. (2001). Comparative genomics of *Listeria* species. *Science* 294 849–852. 11679669 10.1126/science.1063447

[B18] Hardy J. Chu P. Contag C. H. (2009). Foci of *Listeria monocytogenes* persist in the bone marrow. *Dis. Model Mech.* 2 39–46. 10.1242/dmm.000836 19132117 PMC2615163

[B19] Hardy J. Francis K. P. DeBoer M. Chu P. Gibbs K. Contag C. H. (2004). Extracellular replication of *Listeria monocytogenes* in the murine gall bladder. *Science* 303 851–853. 10.1126/science.1092712 14764883

[B20] Joyce S. A. Gahan C. G. (2010). Molecular pathogenesis of *Listeria monocytogenes* in the alternative model host *Galleria mellonella*. *Microbiology* 156(Pt 11), 3456–3468. 10.1099/mic.0.040782-0 20688820

[B21] Karimi S. Ahl D. Vagesjo E. Holm L. Phillipson M. Jonsson H. (2016). In vivo and in vitro detection of luminescent and fluorescent *Lactobacillus reuteri* and application of red fluorescent mCherry for assessing plasmid persistence. *PLOS ONE* 11:e0151969 10.1371/journal.pone.0151969 PMC4803345 27002525

[B22] Kim H. Boor K. J. Marquis H. (2004). *Listeria monocytogenes* σ^B^ contributes to invasion of human intestinal epithelial cells. *Infect. Immun.* 72 7374–7378. 10.1128/IAI.72.12.7374-7378.2004 PMC529113 15557671

[B23] Lauer P. Chow M. Y. Loessner M. J. Portnoy D. A. Calendar R. (2002). Construction, characterization, and use of two *Listeria monocytogenes* site-specific phage integration vectors. *J. Bacteriol.* 184 4177–4186. 10.1128/JB.184.15.4177-4186.2002 12107135 PMC135211

[B24] Lebreton A. Stavru F. Brisse S. Cossart P. (2016). 1926-2016: 90 Years of listeriology. *Microbes Infect.* 18 711–723. 10.1016/j.micinf.2016.10.009 27876526

[B25] Lecuit M. (2007). Human listeriosis and animal models. *Microbes Infect.* 9 1216–1225. 10.1016/j.micinf.2007.05.009 17720601

[B26] Mason E. A. Lopez R. Mason R. P. (2016). Wavelength shifting of chemiluminescence using quantum dots to enhance tissue light penetration. *Opt. Mater. Express* 6 1384–1392. 10.1364/OME.6.001384

[B27] Maury M. M. Tsai Y. H. Charlier C. Touchon M. Chenal-Francisque V. Leclercq A. (2016). Uncovering *Listeria monocytogenes* hypervirulence by harnessing its biodiversity. *Nat. Genet.* 48 308–313. 10.1038/ng.3501 26829754 PMC4768348

[B28] Mead P. S. Slutsker L. Dietz V. McCaig L. F. (2000). Food-related illness and death in the United States. *Emerg. Infect. Dis.* 5 607–625. 10.3201/eid0505.990502 PMC2627714 10511517

[B29] Monk I. R. Casey P. G. Hill C. Gahan C. G. (2010). Directed evolution and targeted mutagenesis to murinize *Listeria monocytogenes* internalin A for enhanced infectivity in the murine oral infection model. *BMC Microbiol.* 10:318 10.1186/1471-2180-10-318 PMC3016325 21144051

[B30] Oozeer R. Furet J. Goupil-Feuillerat N. Anba J. Mengaud J. Corthier G. (2005). Differential activities of four *Lactobacillus casei* promoters during bacterial transit through the gastrointestinal tracts of human-microbiota-associated mice. *Appl. Environ. Microbiol.* 71 1356–1363. 10.1128/AEM.71.3.1356-1363.2005 15746338 PMC1065133

[B31] Park S. F. Stewart G. S. (1990). High-efficiency transformation of *Listeria monocytogenes* by electroporation of penicillin-treated cells. *Gene* 94 129–132. 10.1016/0378-1119(90)90479-B 2121618

[B32] Poulsen K. P. Faith N. G. Steinberg H. Czuprynski C. J. (2011). Pregnancy reduces the genetic resistance of C57BL/6 mice to *Listeria monocytogenes* infection by intragastric inoculation. *Microb. Pathog.* 50 360–366. 10.1016/j.micpath.2011.02.003 21320586 PMC3085720

[B33] Quereda J. J. Dussurget O. Nahori M. A. Ghozlane A. Volant S. Dillies M. A. (2016). Bacteriocin from epidemic *Listeria* strains alters the host intestinal microbiota to favor infection. *Proc. Natl. Acad. Sci. U.S.A.* 113 5706–5711. 10.1073/pnas.1523899113 27140611 PMC4878514

[B34] Rea R. B. Gahan C. G. Hill C. (2004). Disruption of putative regulatory loci in *Listeria monocytogenes* demonstrates a significant role for Fur and PerR in virulence. *Infect. Immun.* 72 717–727. 10.1128/IAI.72.2.717-727.2004 14742513 PMC321596

[B35] Rice B. W. Cable M. D. Nelson M. B. (2001). In vivo imaging of light-emitting probes. *J. Biomed. Opt.* 6 432–440. 10.1117/1.1413210 11728202

[B36] Riedel C. U. Monk I. R. Casey P. G. Morrissey D. O’Sullivan G. C. Tangney M. (2007). Improved luciferase tagging system for *Listeria monocytogenes* allows real-time monitoring in vivo and in vitro. *Appl. Environ. Microbiol.* 73 3091–3094. 10.1128/aem.02940-06 17351089 PMC1892880

[B37] Riedel C. U. Monk I. R. Casey P. G. Waidmann M. S. Gahan C. G. Hill C. (2009). AgrD-dependent quorum sensing affects biofilm formation, invasion, virulence and global gene expression profiles in *Listeria monocytogenes*. *Mol. Microbiol.* 71 1177–1189. 10.1111/j.1365-2958.2008.06589.x 19154329

[B38] Sleator R. D. Watson D. Hill C. Gahan C. G. (2009). The interaction between *Listeria monocytogenes* and the host gastrointestinal tract. *Microbiology* 155(Pt 8), 2463–2475. 10.1099/mic.0.030205-0 19542009

[B39] Szittner R. Jansen G. Thomas D. Y. Meighen E. (2003). Bright stable luminescent yeast using bacterial luciferase as a sensor. *Biochem. Biophys. Res. Commun.* 309 66–70. 10.1016/S0006-291X(03)01530-4 12943664

[B40] Tangney M. Francis K. P. (2012). In vivo optical imaging in gene & cell therapy. *Curr. Gene Ther.* 12 2–11. 10.2174/156652312799789299 22263919

[B41] van Pijkeren J. P. Morrissey D. Monk I. R. Cronin M. Rajendran S. O’Sullivan G. C. (2010). A novel *Listeria monocytogenes*-based DNA delivery system for cancer gene therapy. *Hum. Gene Ther.* 21 405–416. 10.1089/hum.2009.022 20105075

[B42] Van Reet N. Van de Vyver H. Pyana P. P. Van der Linden A. M. Buscher P. (2014). A panel of *Trypanosoma brucei* strains tagged with blue and red-shifted luciferases for bioluminescent imaging in murine infection models. *PLOS Negl. Trop. Dis.* 8:e3054 10.1371/journal.pntd.0003054 PMC4140678 25144573

[B43] Vorobyeva A. G. Stanton M. Godinat A. Lund K. B. Karateev G. G. Francis K. P. (2015). Development of a bioluminescent nitroreductase probe for preclinical imaging. *PLOS ONE* 10:e0131037 10.1371/journal.pone.0131037 PMC4482324 26110789

[B44] Zhao H. Doyle T. C. Coquoz O. Kalish F. Rice B. W. Contag C. H. (2005). Emission spectra of bioluminescent reporters and interaction with mammalian tissue determine the sensitivity of detection in vivo. *J. Biomed. Opt.* 10 41210 10.1117/1.2032388 16178634

