# Supplementary figures and images for: Development of a Click Beetle Luciferase Reporter System for Enhanced Bioluminescence Imaging of Listeria monocytogenes: Analysis in Cell Culture and Murine Infection Models
Source: Front Microbiol. 2017 Sep 26;8:1797. doi: 10.3389/fmicb.2017.01797 (PMC5622934; doi:10.3389/fmicb.2017.01797)

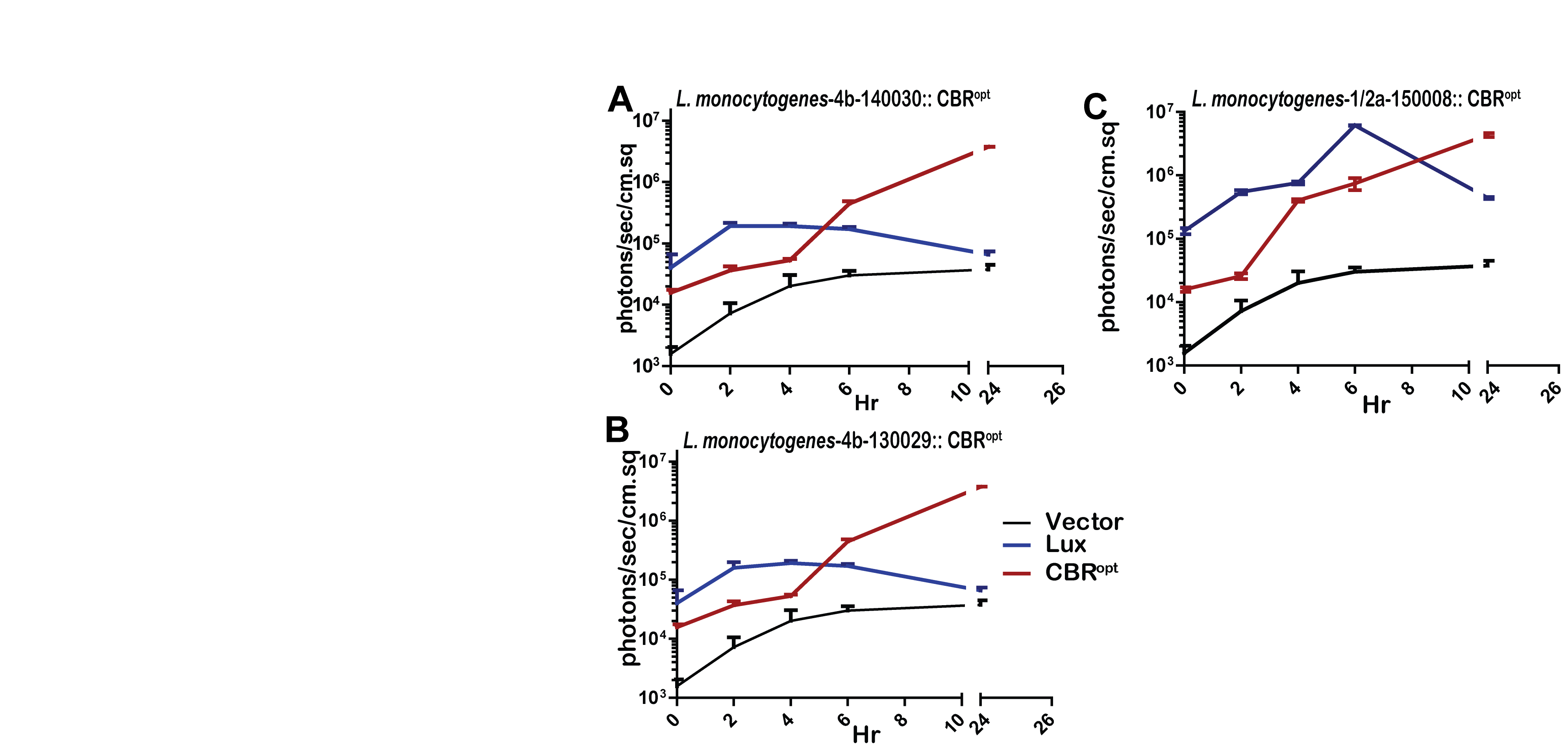

Supplement: FIGURE S1 — Bioluminescence profile of clinical listerial strains labeled with CBRopt reporter. EGDe-4b-130029:pPL2CBRopt, EGDe-4b-140030::pPL2CBRopt, and EGDe-1/2a-150008::pPL2CBRopt (see Table 1) were integrated with the newly developed codon optimized (CBRopt) reporter. The tagged strains were then grown in BHI medium and images were recorded at different time intervals. The overall bioluminescence profile is shown. Data and error bars represent mean and standard deviation, respectively, of triplicate samples for each time point. Asterisks “∗∗” indicates p < 0.05 based upon Student’s t-test. [file Image_1.TIF]

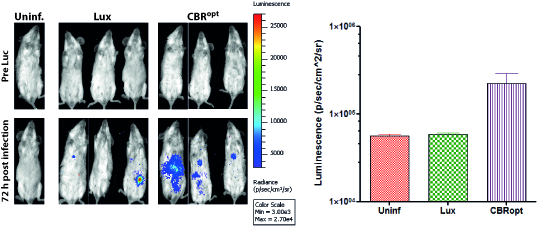

Supplement: FIGURE S2 — In vivo BLI of mice infected with wild type L. monocytogenes EGDe. (A) Whole body luminescence image of BALB/c mice infected with wild type L. monocytogenes EGDe tagged with either bacterial Lux or CBRopt. Mice were infected ip with a dose of 2 × 10^5^ bacteria and were imaged for luminescence at 72 h post-infection. A clear and robust signal is detectable from mice infected with CBRopt tagged bacteria at this timepoint. A luminescent signal that exceeded background (uninfected animals) was detected in every L. monocytogenes EGDe CBRopt infected mouse. The number of CFU/ml recovered from infected mice were not significantly different between both groups (not shown). (B) Graph showing the signal intensity from the whole body images obtained at 72 h from infected mice. Luminescence is shown as total flux (p/s/cm2/sr). Data and error bars represent mean and standard error. [file Image_2.tif]
